# Trajectories of prescription opioids filled over time

**DOI:** 10.1371/journal.pone.0222677

**Published:** 2019-10-04

**Authors:** Jonathan Elmer, Riccardo Fogliato, Nikita Setia, Wilson Mui, Michael Lynch, Eric Hulsey, Daniel Nagin

**Affiliations:** 1 Departments of Emergency Medicine, Critical Care Medicine and Neurology, University of Pittsburgh School of Medicine, Pittsburgh, PA, United States of America; 2 Department of Statistics and Data Science, Carnegie Mellon University, PA, United States of America; 3 Heinz College, Carnegie Mellon University, Pittsburgh, PA, United States of America; 4 Department of Emergency Medicine, Division of Medical Toxicology, University of Pittsburgh School of Medicine, Pittsburgh PA, United States of America; 5 Allegheny County Department of Human Services, Pittsburgh, PA, United States of America; KHANA, CAMBODIA

## Abstract

We performed a retrospective cohort study that aimed to identify one or more groups that followed a pattern of chronic, high prescription use and quantify individuals’ time-dependent probabilities of belonging to a high-utilizer group. We analyzed data from 52,456 adults age 18–45 who enrolled in Medicaid from 2009–2017 in Allegheny County, Pennsylvania who filled at least one prescription for an opioid analgesic. We used group-based trajectory modeling to identify groups of individuals with distinct patterns of prescription opioid use over time. We found the population to be comprised of three distinct trajectory groups. The first group comprised 83% of the population and filled few, if any, opioid prescriptions after their index prescription. The second group (12%) initially filled an average of one prescription per month, but declined over two years to near-zero. The third group (6%) demonstrated sustained high opioid prescriptions utilization. Using individual patients’ posterior probability of membership in the high utilization group, which can be updated iteratively over time as new information become available, we defined a sensitive threshold predictive of sustained future opioid utilization. We conclude that individuals at risk of sustained opioid utilization can be identified early in their clinical course from limited observational data.

## Introduction

Morbidity, mortality and social malady resulting from opioid use disorders are major public health concerns in the United States.[1,2] Prescription opioids were a major driver of the first wave of the opioid epidemic, leading to efforts by legislative bodies, governmental agencies and professional medical societies to limit their inappropriate use.[3–6] Despite potential for misuse, opioids also play an important medical role in the management of pain. Clinicians are thus commonly faced with the challenge of identifying patients or patterns of opioid utilization that may suggest risk for future opioid use disorder and other negative medical, social or legal outcomes.

Providers have several tools at their disposal to help inform judicious prescribing of opioids. Previous observational research has identified demographic factors associated with opioid use disorder risk.[7,8] Prior studies have associated risk of long-term opioid dependence with early opioid prescription characteristics,[9] but these factors have not been translated to patient specific predictive tools. In the chronic pain literature, numerous tools predict medication misuse before initiation of long-term opioid therapy for pain or screen for misuse during long-term management.[10–13] To our knowledge, such opioid-specific tools have not been developed in general medical populations. Finally, prescription drug monitoring programs (PDMPs) and other governmental databases are now available in many states and providers may review patients’ past utilization before issuing a new prescription.[14] These PDMPs are a potentially rich source of longitudinal data that, if modeled appropriately, might inform clinical decision-making by predicting future utilization patterns or abuse potential. Currently, however, PDMPs have no analytical capabilities and only supply providers with raw data.

We used group-based trajectory modeling to identify distinct patterns of prescription opioid utilization among new prescription opioid recipients enrolled in Medicaid in a large county in Southwestern Pennsylvania. Our aims were to identify one or more trajectories comprised of individuals that become chronic, high prescription utilizers; quantify each individual’s time-dependent probability of belonging to a high-utilizer group; and finally to define a sensitive threshold that might trigger clinical intervention before long-term use becomes established.

## Methods

### Setting

Allegheny County is located in western Pennsylvania and includes a population of approximately 1.2 million people. A majority of the population (80%) is Caucasian, with African Americans (13%) and Asians (4%) comprising the two largest minority racial groups. Median age is 41 years and median household income is $54,357. The region has been particularly hard hit by the epidemic of opioid-related overdose and death.[2]

### Data sources and patients

We analyzed a database including individuals living in Allegheny County enrolled in Medicaid between 2009 and 2017 who filled at least one opioid prescription from 2010 to 2017 and received services from the Allegheny County Department of Health and Human Services. Our definition of “opioid” was consistent with those medications falling under the World Health Organization’s Anatomical Therapeutic Chemical N02A subheading, and included weak opioids such as codeine and opioids in combination with other drug classes, excluding opioids in combination with an antagonist such as naloxone. The database is maintained by the Allegheny County Department of Health and Human Services (DHS), and was provided to us in a completely deidentified format for analysis. The database includes details for all filled opioid prescriptions including recipient, medication formulation, route, dose, dispense quantity and duration. These data are linked to individuals’ utilization of DHS programs including child welfare, homelessness, aging, substance use and mental health, as well as data from dozens of external sources that include Medicaid, courts, county jail, medical examiner, birth certificates, public school districts, housing authorities, and state-administered human services.[15] We analyzed all data at a frequency of once per month. Because our interest was in new opioid users, we excluded any subject already receiving opioids in 2009. We further excluded subjects <18 or >45 years of age because indications for chronic opioid therapy in these patients may be distinct.

### Trajectory modeling

Group-based trajectory modeling (GBTM) is a statistical methodology that identifies clusters of individuals following a similar trajectory of one or more measures of interest over time.[16] The statistical details of the methodology have been described in detail elsewhere, particularly in the context of biomedical [17] and sociological research.[18] In the present analysis, we considered each subject’s time 0 (i.e. entry in the trajectory model) to be the month of his or her first opioid prescription. We explored the trajectories of several outcome measures of monthly opioid prescription utilization over four years after entry into the model, or until 2017 after which data were unavailable. First, we considered whether or not any opioid prescription was filled each month as a binary outcome. Second, we considered the total number of prescriptions filled in each month as a count outcome. Finally, we considered the total morphine milligram equivalents (MME) prescribed in each month as a continuous outcome calculated using conversion tables compiled by the Centers for Disease Control and Prevention.[19] We treated these data as following Bernoulli (i.e. logistic), zero-inflated Poisson, and beta distributions, respectively. We scaled MME totals to a 0–1 range by dividing through by the maximum observed value after censoring data at a maximum value of 5000 MME per month to fit the beta distribution. We compared models by visual inspection and Bayesian Information Criteria,[20] and ultimately found the Poisson model to be both best fit and of greatest clinical interest.[21] It was this model we carried forward into subsequent analyses.

### Predictive capabilities

Beyond summarizing the longitudinal patterns of long-term prescription opioid utilization after the index prescription, our particular interest was early identification of individuals at risk of conversion into long-term opioid utilization. An important output of GBTM is each individual’s estimated posterior probability of group membership (PPGM) after each new epoch, in this case each month, based on the observed data to date.[22] To test the predictive capabilities of our model, we trained the Poisson GBTM modeling each subject’s monthly count of opioid prescriptions filled with data from subjects entering the cohort in 2010 or 2011. We then applied this model to two prediction cohorts comprised of subjects entering in 2012–2013 and 2014–2015, and calculated each individual’s monthly PPGM for each trajectory group. For members of the prediction cohorts, we explored various PPGM-based triggers that might serve as sensitive early alarms that the individual in question is at high risk of converting to chronic opioid use. We report the final performance characteristics (true positive, false positive, sensitivity and specificity) of the final PPGM trigger for each of the prediction cohorts.

### Secondary outcomes

After defining distinct trajectories of opioid utilization over time in the training cohort, we compared the association of trajectory group membership with a number of secondary outcomes such as contact with the criminal justice system. Our intent was to place trajectory group membership in the large context of social consequences of long-term opioid use. First, we compared total opioid prescriptions and total MMEs prescribed across trajectory groups in each cohort. Second, because opioid use disorder may predict subsequent contact with the criminal justice system, comorbid mental health disorders, or other social maladies, we compared social and behavioral outcomes across groups. Specifically, we examined the proportion of individuals in each trajectory group that utilized county mental health or substance use disorder services; encountered the child welfare system, those against whom drug-specific criminal charges were filed in county criminal court; and those incarcerated in the county jail. We considered only those outcomes occurring in the years subsequent to the trajectory model membership. Finally, to facilitate comparison to other studies, we calculated the estimated proportion of each trajectory group and the overall population still receiving opioid prescriptions at 12, 24 and 36 months after their index prescription. We explored differences in secondary outcomes across trajectory groups qualitatively to avoid multiple hypothesis testing.

## Results

Overall, 120,650 unique Medicaid recipients in Allegheny County filled at least one opioid prescription from 2009 to 2017 and appeared in the database. Of these, 30,204 filled a prescription in 2009 and 38,990 were not 18–45 years of age leaving 52,456 subjects in the primary analysis (Table 1). Median age of included individuals was 29 [interquartile range (IQR) 22–36] years and 45,994 (61%) were female. Included individuals were most commonly white (46%), with 33% identifying as black or African American, and 18% with no data on race (Table 1). Overall, there were 271,296 opioid prescriptions filled (median 2 [IQR 1–5] prescriptions per individual) for a total of 131,310,760 MMEs (median 285 [IQR 140–725] MME per individual).

**Table 1:** Baseline population characteristics stratified by cohort, as determined by year of index opioid prescription.

Characteristic	Training cohort (2010–11) (n = 18591)	Prediction cohort (2012–13) (n = 11900)	Prediction cohort (2014–15) (n = 10424)
Age, years	28 [22–35]	28 [22–35]	29 [23–3]
Female sex	12916 (69%)	8099 (68%)	6623 (64%)
Race			
White	8692 (47%)	5428 (46%)	4959 (48%)
Black/African American	7044 (38%)	4257 (36%)	3348 (32%)
No Data	2325 (13%)	1805 (15/%)	1788 (17%)
Other	530 (2%)	410 (3%)	329 (3%)
Annual number of opioid prescriptions	0.75 [0.25–1.25]	0.5 [0.25–1.25]	0.5 [0.25–1.00]
MME per prescription	131 [94–200]	130 [90–200]	134 [90–218]
Mental health services	8516 (46%)	4608 (39%)	3580 (34%)
Substance use disorder services	4199 (23%)	2096 (18%)	1578 (15%)
Referred to CYF	2567 (14%)	1230 (10%)	740 (7%)
Criminal charges	6305 (34%)	3107 (26%)	2066 (20%)
Drug charges	2794 (15%)	1298 (11%)	909 (8%)
Incarcerated	3558 (19%)	1768 (15%)	1171 (11%)

Data are presented as median [interquartile range] or number with corresponding percentages. **Abbreviations:** MME–Morphine milligram equivalents; CYF–Office of Children, Youth and Families.

**Fig 1:**
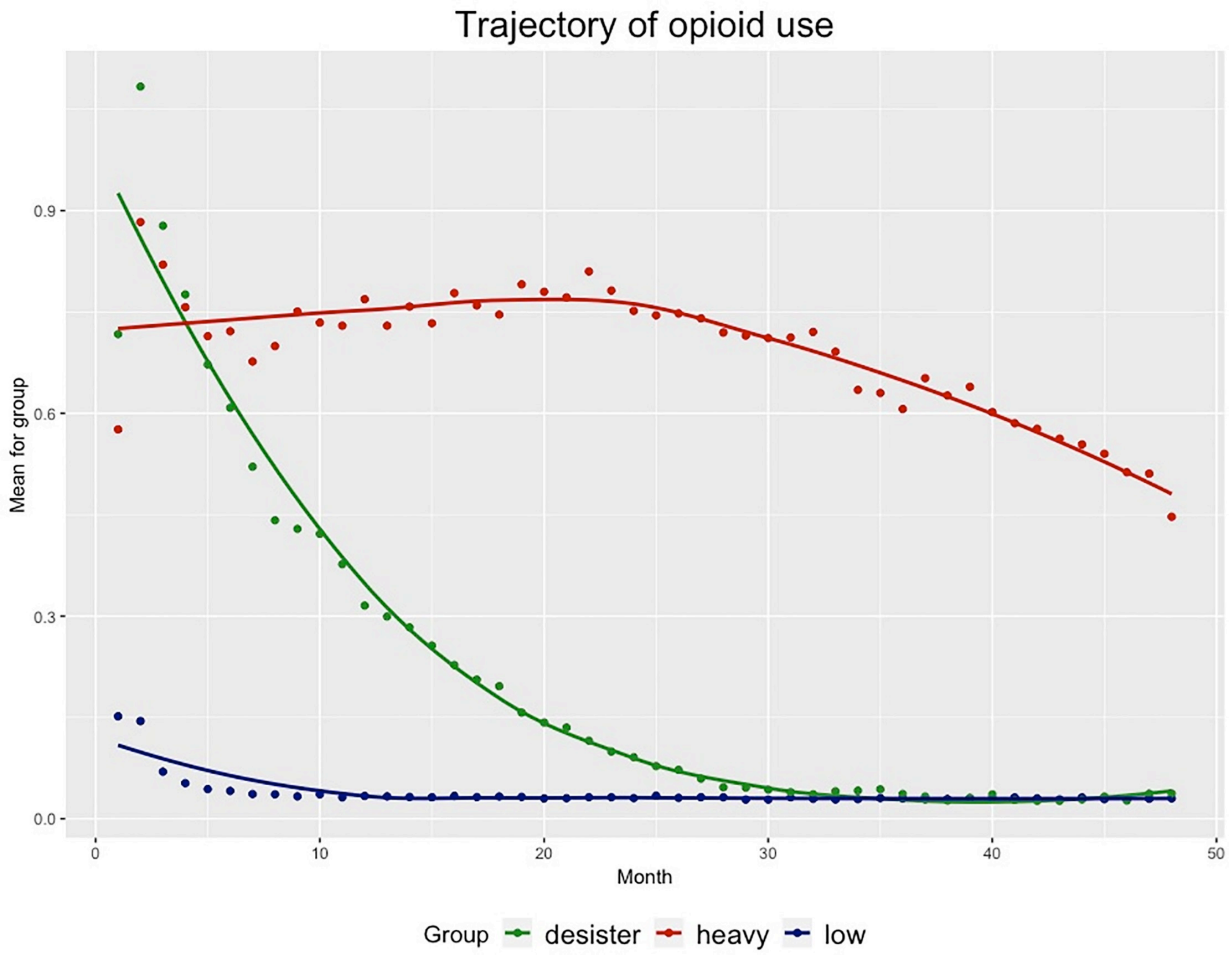
Trajectories of prescription opioid use over a two-year period after the index prescription, modeled in training data from 2010–2011.

In our training cohort, we found the population to be comprised of three distinct trajectory groups (Fig 1 and S1 Table). The first group comprised an estimated 83% of the population filled few, if any, opioid prescriptions after their index prescription. The second group (12%) initially filled an average of one prescription each month, with a gradual decline over two years to near-zero utilization. The third group was a small (6%) but clinically important minority of the cohort with sustained high level of opioid prescriptions filled over the duration of observation. The proportion of individuals belonging to each trajectory group was stable across the prediction cohorts (Table 2). The minority of patients in the sustained high trajectory group filled the bulk of the overall prescriptions in the population (37% of overall prescriptions filled, 61% of overall MMEs dispensed). The majority of individuals filling opioid prescriptions in this cohort were women, with no clear trend in distribution of sex across trajectory groups (Table 3). By contrast, only 45% of the cohort using substance use disorder treatment services was female, and only 52% of those using mental health services were female. There was a stepwise increase in the proportion of individuals identifying as white in higher-utilization trajectory groups. We also observed a stepwise increase in utilization of mental health services across trajectory groups in both the training and prediction cohorts. Contact with other DHS services and the criminal justice system did not systematically vary across groups (Table 3). Overall, 7.6% of the training cohort still received opioid prescriptions 12 months after the index prescription (Table 2). This varied from 2.8% in the sustained low group to 46% in the sustained high group.

**Table 2:** Proportion of the training cohort (2010–11) still receiving opioid prescriptions at 12, 24, and 36 months after their index prescription, stratified by trajectory group.

Time from index prescription	Probability of ongoing receipt of opioid prescriptions			
	Sustained low group (n = 15,452)	Decreasing (n = 2,044)	Sustained high group (n = 1,095)	Overall cohort (n = 18,591)
Month 12	2.8%	23%	46%	7.6%
Month 24	2.5%	7.7%	50%	5.8%
Month 36	2.4%	3.3%	43%	4.8%

**Table 3:** Demographics, opioid prescription characteristics, and social outcomes stratified by cohort and trajectory group.

Characteristic	Training cohort (2010–11) (n = 18,591)			Prediction cohort (2012–13) (n = 11,900)			Prediction cohort (2014–15) (n = 10,424)		
	Group 1 Sustained low	Group 2 Decreasing	Group 3 Sustained high	Group 1 Sustained low	Group 2 Decreasing	Group 3 Sustained high	Group 1 Sustained low	Group 2 Decreasing	Group 3 Sustained high
Age, years	28.3	32.4	34.5	28.0	33.0	35.5	29.0	33.7	35.5
Female sex	70%	62%	69%	69%	60%	65%	65%	55%	61%
Race									
White	44%	62%	64%	43%	60%	70%	45%	61%	68%
Black/African American	40%	27%	27%	38%	26%	21%	34%	24%	19%
No Data	13%	9%	8%	16%	12%	8%	18%	14%	11%
Other	3%	2%	2%	4%	2%	2%	4%	2%	0.0%
Total prescriptions	3.9	13.6	46.3	2.8	11.8	39.1%	2.1	10.3	29.9
MME per prescription	167	463	706	158	404	754	160	411	719
Mental health services	6.6%	10.4%	13.7%	4.6%	7.1%	10.2%	2.9%	4.2%	5.9%
Substance use disorder services	2.6%	4.9%	5.6%	1.8%	3.2%	3.4%	1.3%	2.1%	1.2%
Referred to CYF	2.1%	2.5%	3.4%	1.4%	1.8%	1.6%	0.8%	1.0%	0.6%
Criminal charges	0.9%	1.2%	1.3%	0.6%	0.9%	0.8%	0.4%	0.5%	0.4%
Drug charges	0.3%	0.4%	0.4%	0.2%	0.3%	0.2%	0.1%	0.2%	0.1%
Incarcerated	1.6%	2.3%	1.8%	1.0%	1.7%	1.0%	0.6%	0.8%	0.3%

We identified a PPGM of ≥0.15 for membership in the sustained high trajectory group to be a sensitive threshold to predict potential for long-term prescription opioid use. To minimize false positives, for example an individual ultimately assigned to the declining use trajectory group who initially appeared likely to become a sustained utilizer, we considered an alarm to be triggered any time the PPGM exceeded threshold within a 6-month moving window. Thus, an individual’s alarm flag could be cleared if it became clear after continued observation their behavior more clearly fell into a low-risk trajectory. Operationalized this way, performance characteristics of our alarm flag in each cohort are summarized in Table 4.

**Table 4:** Accuracy metrics of future cohorts, using a 0.15 PPGM cutoff threshold, and a 6-month moving window to identify patients at risk of sustained prescription opioid exposure.

Month	Training cohort (2010–11) (n = 18,591)		Prediction cohort (2012–13) (n = 11,900)		Prediction cohort (2014–15) (n = 10,424)	
	FPR	TPR	FPR	TPR	FPR	TPR
1	0.16	0.359	0.153	0.302	0.152	0.325
2	0.278	0.648	0.26	0.631	0.261	0.712
3	0.324	0.742	0.3	0.748	0.268	0.742
4	0.325	0.745	0.305	0.763	0.279	0.794
5	0.331	0.762	0.31	0.773	0.286	0.829
6	0.338	0.784	0.314	0.794	0.293	0.851
7	0.345	0.799	0.318	0.815	0.296	0.858
8	0.349	0.814	0.322	0.828	0.161	0.811
9	0.188	0.74	0.166	0.761	0.135	0.811

**Abbreviations:** PPGM–Posterior probability of group membership; FPR–False positive rate; TPR–True positive rate.

## Discussion

We used a regional health services dataset to model patient-level trends of opioid prescriptions filled over time. Characterizing the distinct trajectories that describe subpopulations of those receiving prescription opioids is of public health importance as shown by prior work that has linked opioid prescription pattern to risk of overdose, death, opioid misuse and opioid use disorders.[23–25] Most prior analyses of prescription opioid use focus on population-level characteristics. We demonstrate the potential for longitudinal methods to be linked to real-time patient-level risk stratification. We do not propose our model to be the best or only prediction tool that could be developed in this setting. Indeed, the trajectory model we developed was based on a limited subset of the information that is available in PDMPs and it is likely that applying these methods to richer data sets would improve prognostic performance. Such data sets, whether in PDMPs, insurance claims databases or electronic medical records, are already aggregated for other purposes and represent appealing targets for predictive analytics and modeling applications.

For screening or other risk stratification tools to be useful, quantification of an individual’s risk must be tied to a clinical decision or intervention as early in treatment as possible. In our model, we chose to prioritize early sensitivity over specificity, accepting a high proportion of early false positives to achieve a true positive rate >75% after three months of data. As with any predictive model where a threshold is applied to classify patients, sensitivity and specificity can be tuned to align with a particular clinical goal. The sensitive threshold we selected might be appropriately linked to a low risk intervention. For example, at the time a new prescription is ordered, a prescriber could receive an automated prompt encouraging consideration of alternative modes of analgesia or a frank risk/benefit discussion with the prescription recipient. A more specific threshold could allow a more active intervention, for example the decision to not prescribe opioids to an individual or referral to specialty substance use disorder counseling. In our view, the major advantage of GBTM is its ability to identify the likely future trajectory of an individual’s prescription opioid use early, before such a pattern might be obvious to a provider without assistance from a predictive model. In this context, rapidity of available information may be more important to providers than specificity.

Overall, 7.8% of patients in the training cohort still received opioid prescriptions a year after their index prescription. This is consistent with prior work by Shah, et al.[9] Our work adds nuance by allowing classification into distinct trajectory groups with dramatically different use profiles. A majority of individuals filling opioid prescriptions in this cohort identified as white, particularly in higher utilization trajectory groups. This is consistent with national trends in prescription opioid misuse, non-fatal overdose and overdose-related deaths, all of which are proportionally more common among whites than others races.[26] Racial differences across trajectories may also reflect prescribers’ behaviors or biases that result in fewer opioid prescriptions supplied to non-white patients.[27] The preponderance of women filling opioid prescriptions in this population (consistently >60% across trajectory groups and cohorts) is less consistent with national trends in opioid overdose,[26] but consistent with the fact that women report higher levels of prescription opioid (compared to illicit opioid) usage, chronic pain and mood disorders than men.[28,29] The discrepancy between the sex distribution of those filling opioids and those accessing mental health and substance use disorder services merits further exploration in subsequent analyses. In this observational study, we are unable to disentangle the causality by which higher utilization opioid trajectory groups accessed mental health services with increased frequency. Opioid use disorder may lead to both physical and mental health conditions, and vice versa, and the available dataset did not include sufficient detail to support further speculation about the genesis of the observed association. The lack of association between trajectory group and use of substance use disorder services suggests that additional public health efforts may be needed to target available resources to populations at greatest need. Alternatively, it may be that substance use disorder services are only sought when a regular supply of opioids is interrupted, such that patients with sustained high access to prescription opioids do not utilize these services.

Our study has several important limitations. Because clinical data were unavailable, we do not know for what indications patients received their prescriptions. There are undoubtedly medically appropriate, noncontroversial indications for opioid prescription. Patients could fall into the sustained high utilization group because of reasons as divergent as opioid use disorder or bone pain from metastatic cancer with opioids used for palliation at the end of life. Some individuals in this group likely filled these prescriptions for a legitimate, medically necessary indication with appropriate and adherent usage. This limitation is intrinsic to the dataset available to us for analysis, but could be easily overcome in one of two ways. First, more granular data might be modeled using GBTM. Alternatively, in practice GBTM-based risk stratification could simply be interpreted in the context of full available clinical information. Thus, prescribers could be offered a model-based prediction of an individual’s future opioid prescription use pattern and be allowed to decide on a case-by-case basis whether this likely pattern of use is appropriate or inappropriate. For example, any decision support tool might take into consideration not only a patient’s likely future trajectory of prescription opioid use but also the principal diagnosis for which the opioid is being supplied (e.g. cancer-related pain versus chronic low back pain), concomitant diagnoses deemed relevant (e.g. alcohol abuse), etc. Equally importantly, available data are insufficient to assess for non-prescription illicit opioid use, for example heroin, or quantify usage of prescription opioids obtained illegally. Members of any trajectory group may have utilized additional opioids that were not prescribed to them by a medical provider. Similarly, although we know the type and quantity of opioids filled via prescription, we do not know that the individuals filling the prescriptions used the opioids. Some may have been diverted, saved, discarded through appropriate means, or otherwise not used by the prescription recipient. Taken together, these limitations might affect the ability to link trajectory group membership alone to risk of opioid-related negative health or social consequences and demonstrate the need for more robust data for future modeling applications.

The generalizability of our findings is also limited. Available data were derived from a single county’s database of Medicaid recipients and may not represent the general population of the region or Medicaid recipients in other geographic areas. While this limitation is also intrinsic to the dataset we analyzed, it is not a limitation of the modeling methodology. Applied to another dataset, GBTM could yield more broadly generalizable results. Because entry to the cohort began at the index prescription, it may be that some subjects had established chronic prescription opioid use prior to Medicaid eligibility. We used each individual’s index prescription to define time zero in our model, but as in any observational clinical work we do not have knowledge of events that occurred prior to cohort entry. Data were also not available after 2017, so some trajectories in the second prediction cohort were by necessity based on fewer than four full years of data. We observed the vast majority of individuals’ posterior probabilities of group membership to converge on their final values before 24 months of data, so the impact of this censoring on model results was minimal. Finally, we analyzed opioid prescriptions filled at a pharmacy during the study period. Because an individual fills a prescription does not mean that that individual used the prescribed medications. We cannot account for opioids that may have been diverted from prescription recipients to others or use of illicit opioids obtained outside of a pharmacy.

In conclusion, we have used a regional health services dataset to describe trajectories of individuals’ opioid prescriptions over time. We find that a small subgroup comprising just 6% of the population is characterized by sustained high levels of prescription fills. This subgroup accounts for the majority of opioid prescriptions filled over the entire population, and has a significant increase not only in these prescriptions but also potentially related social problems. Group-based trajectory modeling has the potential not just to describe individual prescription patterns over time but also to predict an individual’s likely future behavior. This information can help inform providers and be linked to appropriate clinical interventions to minimize risk to patients.

## Supporting information

S1 Table Trajectory model parameters from the final model. (DOCX)
