# Supplementary material for: Trajectories of prescription opioids filled over time
Source: PLoS One. 2019 Oct 4;14(10):e0222677. doi: 10.1371/journal.pone.0222677 (PMC6777776; doi:10.1371/journal.pone.0222677)
Supplement: S1 Table — (DOCX) [file pone.0222677.s001.docx]

| **Trajectory group** | **Order** | **Coefficient (standard error)** |
| --- | --- | --- |
| Sustained high | Cubic | Intercept: -0.498 (0.020)  Linear: -0.045 (0.019)  Quadratic: 0.032 (0.005)  Cubic: -0.004 (0.0001) |
| Decreasing | Cubic | Intercept: -0.057 (0.016)  Linear: -0.417 (0.024)  Quadratic: -0.079 (0.008)  Cubic: 0.009 (0.001) |
| Sustained low | Intercept-only | Intercept: -0.811 (0.011) |
